# Antithrombotic Management in Patients with Chronic Coronary Syndrome Receiving Oral Anticoagulation

**DOI:** 10.1007/s11886-026-02384-2

**Published:** 2026-06-27

**Authors:** Hubert Dromas, Dany Janah, Basile Verdier, Guillaume Schurtz, Thomas de Saint Nicolas, Gilles Lemesle

**Affiliations:** 1 Intensive Cardiac Care Unit, Heart and Lung Institute, CHU Lille, 59000 Lille, France; 2 Institut Pasteur of Lille, Inserm U1011, 59000 Lille, France; 3 Université de Lille, 59000 Lille, France; 4 French Alliance for Cardiovascular Trials (FACT), Paris, France; 5 Service USIC Et Centre Hémodynamique, Institut Cœur Poumon, Bd du Pr Jules Leclercq, CHU de Lille, 59037 Lille Cedex, France

**Keywords:** Antiplatelet therapy, Oral anticoagulation, Antithrombotics, Chronic coronary syndrome, Percutaneous coronary intervention, Bleeding

## Abstract

**Purpose of Review:**

Atrial fibrillation (AF) and/or the need for oral anticoagulation (OAC) frequently coexist with stable chronic coronary syndrome (CCS). In this population, clinicians must carefully balance ischemic protection against bleeding risk. This review aims to synthesize available evidence and address whether antiplatelet therapy (APT) should be maintained on top of OAC in this specific subset.

**Recent Findings:**

Recent trials have provided key evidence. AFIRE, EPIC-CAD, AQUATIC and ADAPT-AF-DES have all demonstrated that OAC monotherapy is not only non-inferior but also superior to combination therapy (OAC plus APT) in terms of net clinical benefit, with fewer major bleeding events and no increase in ischemic complications. The AQUATIC and AFIRE trials even showed an excess in mortality with prolonged combination therapy.

**Summary:**

Current evidence supports OAC alone as the preferred long-term antithrombotic strategy in patients with AF and stable CCS.

## Introduction

Coronary artery disease (CAD) is the most prevalent cardiovascular disease and remains a leading cause of morbidity and mortality worldwide [1, 2]. The term chronic coronary syndrome (CCS) was first introduced in the 2019 European Society of Cardiology (ESC) guidelines and further refined in the 2024 update to describe the spectrum of clinical presentations of CAD during stable phases [3]. These include patients with a history of acute coronary syndrome (ACS) occurring at least 1 year prior, those with stable angina, and/or those with previous coronary revascularization at least 1 year prior. Following ACS or percutaneous coronary intervention (PCI), dual antiplatelet treatment (DAPT) is recommended for 6 to 12 months to reduce the risk of stent thrombosis and recurrent ischemic events [3–6]. Thereafter, long-term single antiplatelet therapy (SAPT), using either aspirin or clopidogrel, is recommended in patients with CCS to prevent recurrent atherothrombotic complications [3, 6, 7].

Importantly, in clinical practice, approximately 15% of patients with CCS also require long-term oral anticoagulation (OAC), most commonly due to atrial fibrillation (AF) [8, 9]. Other indications include left ventricular thrombus, mechanical valves or venous thromboembolic disease. OAC use is associated with an increased bleeding risk, particularly when combined with antiplatelet therapy [10–12]. However, concerns regarding recurrent ischemic events, including stent thrombosis or ACS, have historically supported the continuation of combined antithrombotic therapy with OAC and SAPT [9, 13–15]. Until recently, the optimal long-term antithrombotic strategy for patients receiving OAC (mainly for AF) with stable CCS remained uncertain. Although international guidelines have recommended OAC monotherapy in this setting, the level of supporting evidence was limited, particularly in patients at high risk of recurrent ischemic events and/or with complex coronary anatomy.

Several key recent randomized controlled trials (RCTs) have helped address this important question in clinical practice. The aim of the present review is to summarize recent evidence and clarify the optimal antithrombotic strategy in patients with CCS requiring OAC.

### Rationale for Antithrombotic Strategies in Chronic Coronary Syndrome

Platelet activation and aggregation, leading to thrombus formation, are key mechanisms in the pathogenesis of CAD, both in ACS and CCS [16, 17]. Accordingly, in addition to risk factor control, antiplatelet therapy remains the cornerstone of thrombotic event prevention in CCS [3].

Aspirin monotherapy was the first antithrombotic strategy to demonstrate its efficacy to reduce mortality and ischemic events in patients with CCS. These benefits outweigh the modest increase in major gastrointestinal and extracranial bleeding from 0.07 to 0.1% per year [7, 18]. More recently, clopidogrel monotherapy has also been shown to reduce mortality and major adverse cardiovascular events (MACE) in this population, making it a viable alternative to aspirin for long-term antithrombotic treatment [3, 19, 20]. It has even been shown to be superior to aspirin in the HOST-EXAM trial [19].

After PCI or ACS, DAPT with aspirin and a P2Y12 inhibitor significantly reduces the risk of stent thrombosis and myocardial infarction (MI) compared with aspirin alone and is recommended for 6 to 12 months [3, 5, 6]. However, the duration of DAPT may considerably vary according to patient subsets. The DAPT trial demonstrated that prolonging DAPT beyond 1 year reduced late stent thrombosis and MACE compared with aspirin monotherapy, but at the cost of an increased risk of moderate and severe bleeding [21]. Conversely, the MASTER-DAPT trial showed that shortening DAPT to 1 month after PCI in high bleeding-risk patients was non-inferior to 3–6 months with respect to net clinical outcomes [22]. Therefore, selected patients at high residual ischemic risk may benefit from prolonged DAPT while patients at high bleeding risk must benefit from shorter DAPT duration.

Overall, in patients with CCS who do not require long-term OAC, aspirin monotherapy remains the standard long-term strategy to reduce the risk of MI, stroke and vascular death, although clopidogrel monotherapy represents a reasonable alternative according to recent guidelines and trial results [3, 7].

### Rationale for Antithrombotic Strategies in Atrial Fibrillation

Endothelial dysfunction, hypercoagulability, and intra-atrial blood stasis promote thrombus formation in AF. The risk of thromboembolic event and particularly ischemic stroke is increased fivefold compared with the general population [23, 24]. International guidelines recommend long-term OAC for stroke prevention in AF [25].

Vitamin K antagonists (VKAs) have been shown to be superior to aspirin monotherapy or DAPT for stroke prevention in patients with AF [26]. Direct oral anticoagulants (DOACs, i.e.: apixaban, rivaroxaban, edoxaban and dabigatran) have demonstrated either non-inferiority or superiority compared with VKA in this indication. They have also been shown to provide lower rates of intracranial bleeding and, in some trials, reduced mortality [27]. Currently, they are therefore the preferred anticoagulants because of their favourable safety profile and ease of use, without the need for routine laboratory monitoring [25].

### CCS and the Need for Long-Term OAC: A Frequently Encountered Clinical Situation

AF represents the main indication for long-term OAC among patients with CCS. In clinical practice, up to 15% of patients with CCS require long-term OAC for various reasons. On the other hand, approximately 30% of AF patients also have concomitant CCS [9, 28–30]. These two conditions share several common risk factors such as age, hypertension, diabetes and obesity [25]. In addition, CAD and history of MI are also associated with ischemic cardiomyopathy and adverse left ventricular and atrial remodelling, thereby leading to a higher risk of AF development over time [31].

### Balancing Bleeding and Ischemic Risks: Observational Data

Importantly, observational data have consistently shown that patients with AF and CCS are at high risk of both ischemic and bleeding events, making antithrombotic management particularly challenging in this specific population [8]. The absence of dedicated RCT in this field has historically complicated the selection of the optimal antithrombotic strategy for clinicians.

In the international CLARIFY and REACH registries, AF was associated with a twofold increase in the composite endpoint of cardiovascular death, MI or stroke compared with CCS patients without AF [9, 32]. In addition, the risk of major bleeding was also increased 4.5-fold among patients with AF [32]. Similarly, in the French CORONOR registry, CCS patients with OAC had a threefold higher incidence of MACE (6.5% per year) and a 4.7-fold higher incidence of major bleeding (2.5% per year) compared with patients not receiving OAC [10].

Although bleeding events might usually be underestimated compared with ischemic events, they are associated with a substantial mortality. In CORONOR, major bleeding events (BARC ≥ 3) were half as frequent as major ischemic events (3.1% vs 6.3%), yet were associated with a threefold higher mortality [33]. Moreover, the occurrence of major bleeding after ACS or PCI increases the risk of death by 2- to fivefold [34–38].

Several mechanisms may explain this adverse prognosis. First, there is an overlap between risk factors for ischemic and bleeding events, including but not limited to advanced age, diabetes mellitus and chronic kidney disease [10, 32, 33]. Second, bleeding events may directly contribute to worse outcomes through hypotension, anaemia, and tissue hypoxemia, which have been associated with increased mortality in CCS patients [39, 40]. In addition, bleeding frequently leads to premature discontinuation of antithrombotic therapy, thereby increasing the risk of recurrent ischemic events. Finally, consequent blood transfusions have also been associated with higher mortality. Pathophysiological hypotheses include platelet hyperactivation, vasoconstriction, and nitric oxide pathway alterations [36].

Notably, registry data highlight substantial heterogeneity in antithrombotic management. In the REACH registry, only 52% of AF patients received OAC, and 16.3% received both OAC and antiplatelet therapy [9]. In the GARFIELD-AF registry, 14% of patients were treated with dual therapy (OAC plus antiplatelet therapy) [13, 14]. In CLARIFY, OAC alone was used in 25.7% of patients, antiplatelet therapy alone in 52.8% and both in 21.5% [15].

This heterogeneity in clinical practice underscores the historical uncertainty regarding the optimal antithrombotic strategy in patients with CCS and AF.

Whether SAPT (either aspirin or clopidogrel) should be maintained in addition to OAC, especially in high-risk patients, or OAC monotherapy is sufficient therefore requires careful assessment of the balance between ischemic and bleeding risks. Adequately powered RCTs were needed to establish evidence-based recommendations in this challenging clinical setting.

### Optimal Antithrombotic Regimen in Patients with CCS Receiving OAC: Final Answer From Randomized Controlled Trials

Six pivotal RCTs have evaluated OAC monotherapy versus combination therapy (OAC plus SAPT) in patients with CCS requiring long-term OAC. Their main characteristics are summarized in Table 1, and outcomes in Table 2.

**Table 1:** Key characteristics of the 6 randomized clinical trials comparing OAC monotherapy versus combination therapy with antiplatelet agents in patients with stable chronic coronary syndrome receiving OAC

Trial	Year of publication	Design	Population	Number of participants	Antithrombotic strategies	OAC used	Median follow-up
OAC-ALONE	2018	Non-inferiority Open-label	Asian AF + CCS with PCI > 1 year	690	OAC monotherapy *vs* OAC + SAPT: Aspirin 81–324 mg o.d. (86%) *or* Clopidogrel 75 mg o.d. (14%)	VKA (75%) Apixaban (10%) Rivaroxaban (6%) Dabigatran (6%) Edoxaban (3%)	30 months
AFIRE	2019	Non-inferiority Open-label	Asian AF + CCS (PCI/CABG ≥ 1 year or unrevascularized CAD)	2215	Rivaroxaban monotherapy *vs* Rivaroxaban + SAPT: Aspirin 81–100 mg o.d. (70%) *or* Clopidogrel 50–75 mg o.d. (25%) *or* Prasugrel (2%)	Rivaroxaban	24.1 months
PRAEDO-AF	2022	Non-inferiority Open-label	Asian AF + CCS with PCI/CABG ≥ 6 months)	147	Edoxaban monotherapy *vs* Edoxaban + Clopidogrel 75 mg o.d	Edoxaban	20.8 months
EPIC-CAD	2024	Superiority Open-label	Asian AF + CAD with PCI/CABG ≥ 6 months or ACS ≥ 12 months or stenosis ≥ 50%	1040	Edoxaban monotherapy *vs* Edoxaban + SAPT: Aspirin (62%) *or* Clopidogrel (38%)	Edoxaban	12 months
AQUATIC	2025	Superiority Double-blind Placebo-controlled	European OAC (89% for AF) + CCS with PCI > 6 months and high residual atherothrombotic risk	872	OAC + placebo *Vs* OAC + Aspirin 100 mg o.d	VKA (10%) Apixaban (62%) Rivaroxaban (25%) Dabigatran (3%)	26.4 months
ADAPT-AF-DES	2025	Non-inferiority Open-label	Asian AF + CCS with PCI ≥ 1 year	960	OAC monotherapy *vs* OAC + Clopidogrel 75 mg o.d	Apixaban (65%) Rivaroxaban (35%)	12 months

*ACS* acute coronary syndrome; *AF* atrial fibrillation; *CABG* coronary artery bypass grafting; *CAD* coronary artery disease; *CCS* chronic coronary syndrome; *OAC* oral anticoagulant; *o.d.* once a day; *PCI* percutaneous coronary intervention; *SAPT* single antiplatelet treatment; *VKA* vitamin K antagonist

**Table 2:** Main results of the 6 randomized trials comparing oral anticoagulant monotherapy versus combination therapy with antiplatelet agents in patients with CCS receiving OAC

Trial	Primary endpoint	Main outcome	Mortality	Ischemic events*	Stent thrombosis	Major bleeding	Interpretation
OAC-ALONE	Death, MI, stroke, systemic embolism	15.7% (monotherapy) vs 13.6%	11.6% vs 9%	10.5% vs 9%	0.5% vs 0%	7.8% vs 10.4%	Non-inferiority not met (underpowered)
AFIRE	Stroke, systemic embolism, MI, unstable angina requiring revascularization, death	4.1% per patient-year (monotherapy) vs 5.8%	3.7% vs 6.6%	5.4% vs 6.8%	No available data	3.2% vs 5.2%	Rivaroxaban monotherapy is safer than combination therapy
PRAEDO-AF	Major bleeding, clinically significant bleeding	1.67% per patient-year (monotherapy) vs 4.28%	4% vs 1.3%	1.4% vs 0%	0	0	Inconclusive (underpowered)
EPIC-CAD	Death, MI, stroke, systemic embolism, unplanned urgent revascularization, major bleeding, clinically relevant nonmajor bleeding	6.8% (monotherapy) vs 16.2%	0.6% vs 0.7%	3% vs 2.4%	0	1.3% vs 4.5%	Edoxaban monotherapy is better than combination therapy
AQUATIC	Cardiovascular death, MI, stroke, systemic embolism, coronary revascularization, acute limb ischemia	12.1% (placebo) vs 16.9% (aspirin)	8.4% vs 13.4%	9.1% vs 10.6%	0.2% in both groups	3.4% vs 10.2%	OAC monotherapy is better than combination therapy with Aspirin
ADAPT-AF-DES	Death, MI, stent thrombosis, stroke, systemic embolism, major bleeding	9.6% (monotherapy) vs 17.2%	2.9% vs 4%	3.3% vs 4%	0.4% in both groups	2.3% vs 6.1%	Apixaban or Rivaroxaban monotherapy is better than combination therapy with Clopidogrel

Results are displayed as percentage in the OAC monotherapy group (placebo group for the AQUATIC trial) vs percentage in the combination group (aspirin group for the AQUATIC trial)

*MI* myocardial infarction; *OAC* oral anticoagulant

^*^ Definition depends on the study:

- OAC-ALONE: composite of cardiovascular death, myocardial infarction, ischemic stroke, or systemic embolism

- AFIRE: composite of death from any cause, myocardial infarction, unstable angina requiring revascularization, stroke, transient ischemic attack, systemic arterial embolism, venous thromboembolism, revascularization or stent thrombosis

- PRAEDO-AF: one event was recorded for the composite of ischemic or systemic stroke and cardiovascular death. No events were recorded for the following outcomes: myocardial infarction, stent thrombosis, or unstable angina requiring revascularization

- EPIC-CAD: composite of any ischemic events was defined post hoc as a composite of death from any cause, myocardial infarction, ischemic stroke, systemic embolism, or unplanned urgent revascularization

- AQUATIC: an atherothrombotic cardiovascular event was a composite of myocardial infarction, stent thrombosis, stroke, coronary revascularization, systemic embolism or acute limb ischemia

- ADAPT-AF-DES: composite of cardiovascular death, myocardial infarction, stent thrombosis, stroke, or systemic embolism

OAC-ALONE was the first trial addressing this question but was prematurely terminated due to slow enrolment and was markedly underpowered, therefore precluding any definite conclusions [12].

The AFIRE trial subsequently demonstrated non-inferiority of rivaroxaban monotherapy compared with combination therapy for the composite endpoint of death from any cause, stroke, systemic embolism, MI, or unstable angina requiring revascularization. Of note, this trial was stopped early because of excessive mortality in the combination therapy group. Major bleeding was significantly reduced with monotherapy [11]. However, this trial primarily included low-risk patients, and 30% had no history of previous stent implantation.

PRAEDO-AF and EPIC-CAD both evaluated edoxaban-based strategies (edoxaban monotherapy versus edoxaban plus SAPT). PRAEDO-AF was severely underpowered (n = 147 patients) [41]. Edoxaban monotherapy was superior to combination therapy for the composite endpoint of death from any cause, MI, stroke, systemic embolism, unplanned urgent revascularization, major bleeding or clinically relevant nonmajor bleeding in EPIC-CAD. This result was mainly driven by a lower incidence of bleeding events in the monotherapy group [42]. Similarly, EPIC-CAD included low-risk patients and a substantial proportion (40%) had no prior stent implantation. Notably, only 35% of patients in AFIRE and 15% in EPIC-CAD had a history of MI [11, 42].

In 2025, two new key trials were published, further strengthening the evidence, and enrolled exclusively patients with a history of PCI. The AQUATIC trial was the first, and only, double-blinded RCT to compare OAC monotherapy (plus placebo) with OAC plus aspirin in CCS patients. Importantly, it included patients at high risk of ischemic events (enrichment criteria) with an indication for long-term anticoagulation. Similar to AFIRE, the study was stopped early due to excess mortality in the aspirin group. It showed an increase in the risk of a composite of cardiovascular death, MI, stroke, systemic embolism, coronary revascularization, and acute limb ischemia in the combination group (OAC plus aspirin) compared with OAC monotherapy (placebo group). Major bleeding events and all-cause mortality were lower in the placebo group [43]. The ADAPT-AF-DES compared OAC monotherapy with combination therapy (OAC plus clopidogrel). Monotherapy was non-inferior to combination therapy for the composite of death from any cause, MI, stent thrombosis, stroke, systemic embolism, or major bleeding or clinically relevant nonmajor bleeding [44, 45]. Of note, 72% of AQUATIC patients had a history of MI and 64% of ADAPT-AF-DES had a history of ACS, highlighting the high ischemic risk of these patients [43, 44].

Across trials, ischemic event rates were similar between strategies, whereas bleeding reduction consistently favoured OAC monotherapy (Table 2). Recent meta-analyses pooling the results from all these RCTs confirmed that combination therapy (OAC plus SAPT) significantly increases major bleeding without reducing ischemic events (nor stent thrombosis, MI or stroke). In addition, combination therapy was associated with a non-significant trend toward higher mortality [46, 47].

### Clinical Implications and Guidelines Perspectives

In light of the recent evidence, OAC monotherapy should be favoured in patients with CCS requiring long-term OAC.

Current European and American guidelines recommend a short course of triple therapy (aspirin, P2Y12 inhibitor, and OAC) for 1 to 4 weeks after PCI, followed by dual therapy (P2Y12 inhibitor plus OAC) for up to 12 months [3, 6]. Beyond 1 year, switching to OAC monotherapy has been recommended, although the supporting level of evidence was previously limited. The results of recent RCTs subsequently strengthen the evidence base supporting OAC monotherapy as the optimal antithrombotic strategy in this setting. Across trials, monotherapy consistently reduced bleeding events without increasing ischemic outcomes, including in patients at high ischemic risk (as shown in the AQUATIC trial) [43].

**Fig. 1:**
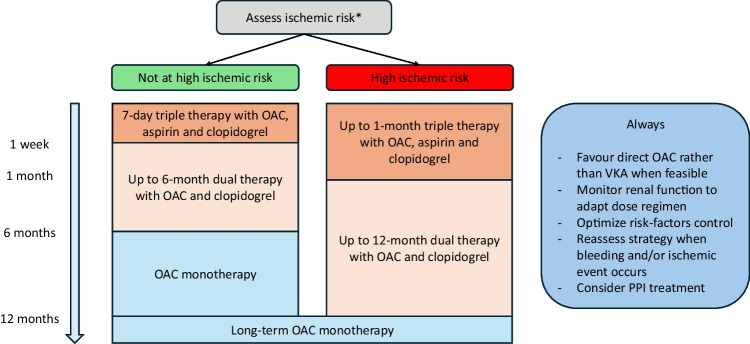
Proposed antithrombotic management algorithm for patients receiving oral anticoagulation after percutaneous coronary intervention. *Assess ischemic risk according to the European Society of Cardiology 2024 guidelines, ischemic risk depends on anatomical, procedural and clinical characteristics. Ischemic risk factors include stenting of left main stem, proximal left anterior descending artery, last remaining patent artery, suboptimal stent deployment, stent length > 60 mm, diabetes mellitus, chronic kidney disease, bifurcation with 2 stents implanted, treatment of chronic total occlusion, and previous stent thrombosis on adequate antithrombotic therapy. OAC: oral anticoagulation; VKA: vitamin K antagonist; PPI: proton pump inhibitors

Therefore, discontinuation of all antiplatelet therapy (either aspirin or clopidogrel) beyond 1 year after PCI appears appropriate in CCS patients receiving OAC, in the absence of another clear indication for antiplatelet therapy treatment. The Fig. 1 depicts how antithrombotics should be managed in this setting.

### Unresolved Issues and Knowledge Gaps

Despite the major advances provided by recent RCTs, several issues remain unresolved.

First, most trials evaluated dual strategies combining anti-Xa OAC (apixaban, rivaroxaban or edoxaban) with aspirin or clopidogrel (Table 1). Anti-Xa agents currently represent the most widely used OAC in clinical practice. Data for VKA and dabigatran in this specific long-term setting remain limited. Therefore, extrapolation to these agents should be undertaken with caution. Nevertheless, given the well-established bleeding risk associated with combination therapy, VKA monotherapy may reasonably be preferred over continued dual therapy in stable CCS patients. Considering new DOACs (factor XI inhibitors) currently under investigation, further studies will be needed to clarify the optimal antithrombotic strategy if they are approved [48, 49].

Second, most RCTs were conducted in Asian populations, except for AQUATIC, which may limit generalizability to western populations. Differences in CYP-mediated metabolism, particularly affecting clopidogrel, as well as variations in DOAC and antiplatelet dosing regimens (Table 1), may influence treatment effects.

Third, the incidence of stent thrombosis was very low across trials (Table 2). Although this event has become very rare in the modern drug eluding stents era, follow-up duration may not have been sufficient to fully assess very late thrombotic events (Table 1). In addition, several trials were terminated early, which may have led to overestimation of treatment effects.

Fourth, the optimal timing for discontinuation of all antiplatelet therapy after PCI or ACS in patients receiving OAC remains uncertain. In most RCTs, the median time from the most recent PCI to enrolment ranged from 3 to 4.5 years. Post-hoc subgroup analyses from AQUATIC suggest that switching to OAC monotherapy as early as 6 months after PCI may be safe, although this hypothesis requires prospective validation [43]. The ongoing RCT MATRIX-2 trial (NCT05955365) is investigating a strategy of discontinuing all antiplatelet therapy 1 month after PCI in patients receiving OAC, and may provide further insight into earlier de-escalation strategies [50].

Fifth, in CCS patients receiving OAC alone, how the antithrombotic regimen should be managed in the event of temporary OAC discontinuation for an invasive procedure or surgery remains unclear and largely depends on the duration of discontinuation. Basically, 3 strategies may be considered: 1- stop OAC without any bridging, 2- stop OAC and temporarily switch to aspirin and 3- stop OAC and temporarily switch to intravenous or subcutaneous heparin.

Finally, AF was the indication for anticoagulation in the vast majority of included patients (Table 1). This is consistent with observational data, as AF represents the most frequent indication for OAC prescription in this population. However, data remain extremely limited for other indications such as mechanical valves, left ventricular thrombus, or venous thromboembolic disease. AQUATIC was the only trial to include patients with indications for OAC other than AF, and these patients represented only 11% of the study population [43]. In the absence of dedicated randomized evidence, management decisions in these populations must rely on extrapolation and individualized risk assessment.

## Conclusions

Data from several recent RCTs have strengthened the evidence supporting current guideline recommendations. In patients with CCS receiving long-term OAC, combination therapy with OAC and antiplatelet agents is associated with a higher risk of bleeding and increased mortality compared with OAC alone, without reducing ischemic events. Accordingly, OAC monotherapy should be the preferred long-term antithrombotic strategy in these specific patients. Routine continuation of aspirin or clopidogrel should generally be avoided in this population in the absence of another clear indication. The optimal timing for discontinuation of all antiplatelet therapy after PCI, as well as the role of new emerging antithrombotic strategies and novel agents, warrants further investigations.

## Key References

- Lemesle G, Didier R, Steg PG, Simon T, Montalescot G, Danchin N, et al. Aspirin in Patients with Chronic Coronary Syndrome Receiving Oral Anticoagulation. N Engl J Med. 2025;393:1578‑88. 10.1056/NEJMoa2507532.
  - First double-blind placebo-controlled randomized trial demonstrating that the addition of aspirin to oral anticoagulation increases adverse clinical outcomes, mortality and major bleeding compared with oral anticoagulation monotherapy in patients with chronic coronary syndrome, previous coronary stent (> 6 months), and high residual ischemic risk.
- Lee S-J, Yu HT, Lee Y-J, Lee S–H, Heo JH, Ahn SG, et al. Therapy for Atrial Fibrillation in Patients with Drug-Eluting Stents. N Engl J Med. 2026 Feb 12;394(7):658–668. 10.1056/NEJMoa2512091.
  - Open-label randomized controlled trial showing non-inferiority of oral anticoagulation monotherapy compared with oral anticoagulation plus clopidogrel in chronic coronary syndrome beyond 1 year after coronary stent implantation.
- Garagoli F, Masson W, Lobo M, Barbagelata L, Cayla G, Gilard M, et al. Antiplatelet therapy in patients with chronic coronary syndrome requiring oral anticoagulation: An updated meta-analysis of randomized trials. Current Probl Cardiol. 2026 Mar;51(3):103250. 10.1016/j.cpcardiol.2025.103250.
  - Recent meta-analysis incorporating the results of AQUATIC and ADAPT-AF-DES, confirming that oral anticoagulation monotherapy reduces major bleeding without increasing ischemic events compared with combination therapy.

## Data Availability

No datasets were generated or analysed during the current study.
